# Polyphenols as Potential β-Lactamase Inhibitors: An Integrated Computational and Experimental Study

**DOI:** 10.3390/molecules30224416

**Published:** 2025-11-15

**Authors:** Fatima Mourabiti, Fatimazahra Jouga, Lorena G. Calvo, Rosa-Antía Villarino, Yassine Zouheir, Abdelaziz Soukri, Trinidad de Miguel, Bouchra El Khalfi

**Affiliations:** 1 Laboratory of Health, Environment and Biotechnology, Team of Physiopathology, Molecular Genetics and Biotechnology, Faculty of Sciences Ain Chock, Hassan II University of Casablanca, Casablanca 20100, Morocco; fatimaamourabiti@gmail.com (F.M.); jougafatimazahra@gmail.com (F.J.); a_soukri@hotmail.com (A.S.); bouchra.elkhalfi@gmail.com (B.E.K.); 2 Department of Microbiology and Parasitology, Universidade de Santiago de Compostela, E-15782 Santiago de Compostela, Spain; lorena.gomez.calvo@rai.usc.es (L.G.C.); rosaantia.villarino@rai.usc.es (R.-A.V.); 3 Laboratory of Molecular Bacteriology, Pasteur Institute, Casablanca 20250, Morocco; yassine.zouheir@pasteur.ma

**Keywords:** carbapenemases, in silico, molecular docking, Gram-negative bacteria, β-lactamases inhibition, antibiotic potentiation

## Abstract

The production of β-lactamases is the main mechanism underlying carbapenem resistance. This study combined in silico and in vitro approaches to identify potential polyphenols as carbapenemase inhibitors. Molecular docking, molecular dynamics, and ADMET prediction were performed to assess the binding affinity, stability, and safety of quercetin, kaempferol, caffeic acid, and 3,4-dihydroxybenzoic acid against KPC-2, NDM-1, and OXA-48 carbapenemases. In vitro antibacterial assays and checkerboard analyses were conducted against *Klebsiella pneumoniae*, *Escherichia coli*, and *Pseudomonas aeruginosa* to assess antibacterial and synergistic effects. Then, the inhibition of the β-lactam hydrolytic activity was confirmed. In silico results showed that quercetin, kaempferol, and caffeic acid exhibited strong binding affinity and consistent stability towards the targets. Therefore, quercetin and kaempferol showed the strongest affinities (−8.0 kcal/mol) and stable interactions with key catalytic residues. ADMET profiles indicated good pharmacokinetic behavior and low acute toxicity. In vitro assays revealed that the polyphenols exhibited MIC values ranging from 12.5 to 25 mg/L and MBC values of 25–50 mg/L. Combined with cefotaxime, they enhanced bacterial susceptibility and inhibited β-lactam hydrolysis, with quercetin achieving complete inhibition at 200 mg/L. These findings highlight the potential of the four polyphenols as natural β-lactamase inhibitors. Further enzyme kinetics and in vivo studies are needed to confirm their therapeutic relevance.

## 1. Introduction

**Figure 1:**
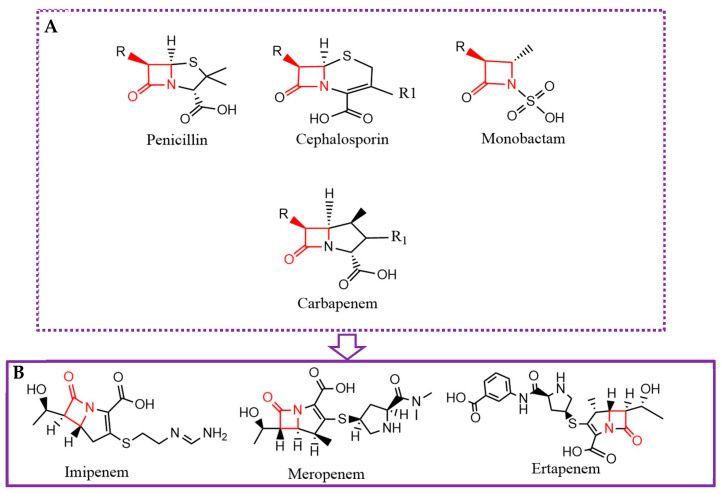
General chemical structure of β-lactam antibiotics highlighting the conserved β-lactam ring and variable side chains that define each subclass (**A**). Detailed structure of the most relevant carbapenems, including Imipenem (**B**) (ChemDraw v21.0.0).

β-lactams are frequently utilized in clinical practice, but they are susceptible to neutralization by an increasing emergence of β-lactamases. The β-lactam ring (Figure 1) of this class of antibiotics is degraded by the β-lactamase enzyme, preventing antibiotics from binding to their drug targets [1]. Carbapenems are the most potent antibiotics among β-lactams in treating infections due to multidrug-resistant (MDR) bacteria. Consequently, due to their safety and efficacy, they are broadly used. This has led to the rise in carbapenem resistance, illustrating a major global public health problem [2]. Currently, carbapenem-resistant pathogens, including *Enterobacteriaceae* and *P. aeruginosa*, were mentioned by the World Health Organization (WHO) in 2024 as the priority pathogens, for which it is crucial to develop novel drugs [3]. The deficiency of effective treatments for carbapenem-resistant *Enterobacteriaceae* infections leads to mortality rates as high as 40 to 50%, which impacts the financial costs of patients’ hospitalization and threatens the most important medical achievements of the last century [2]. The development of carbapenemase inhibitors is a cost-effective and fast strategy to restore the sensitivity to carbapenems, compared with the development of new drugs [4]. The development and discovery of promising therapeutic approaches that provide new methods of treatment are winning huge interest, due to the urgent need for innovative alternatives [3].

According to ethnobotanical data, plants could be an alternative source of antibacterial compounds. The antibacterial action of medicinal plants lies in the presence of bioactive compounds, such as quinolones, flavonoids, phenols, terpenoids, and alkaloids [5]. In recent years, natural products have been reported to have antimicrobial effects as well as various therapeutic benefits. In addition, polyphenols attract global attention due to their high antimicrobial activity. It has also been reported that some polyphenols have synergistic effects when combined with other polyphenols or antibiotics [6].

The current study aims to elucidate the inhibitory potential of quercetin, kaempferol, caffeic acid, and 3,4-dihydroxybenzoic acid using both computational and experimental approaches. Therefore, we have employed molecular docking, molecular dynamics simulations, and ADMET prediction to evaluate their binding, stability, and safety profiles. Furthermore, we have experimentally assessed their antibacterial activity against carbapenem-resistant and sensitive Gram-negative bacteria, including *Klebsiella pneumoniae*, *Escherichia coli*, and *Pseudomonas aeruginosa*, characterized as carbapenemase-producing strains involved in human nosocomial infections. Additionally, we evaluated the combined effect of these polyphenols with a β-lactam antibiotic and explored their mechanism of β-lactamase inhibition. By integrating all in silico predictions with in vitro validation, this study addresses a clear knowledge gap by providing a comprehensive assessment of these polyphenols as potential β-lactamase inhibitors, highlighting novel therapeutic avenues to combat infections caused by β-lactamase-producing bacteria.

## 2. Results

### 2.1. Computational Study

#### 2.1.1. Molecular Docking Analysis

**Figure 2:**
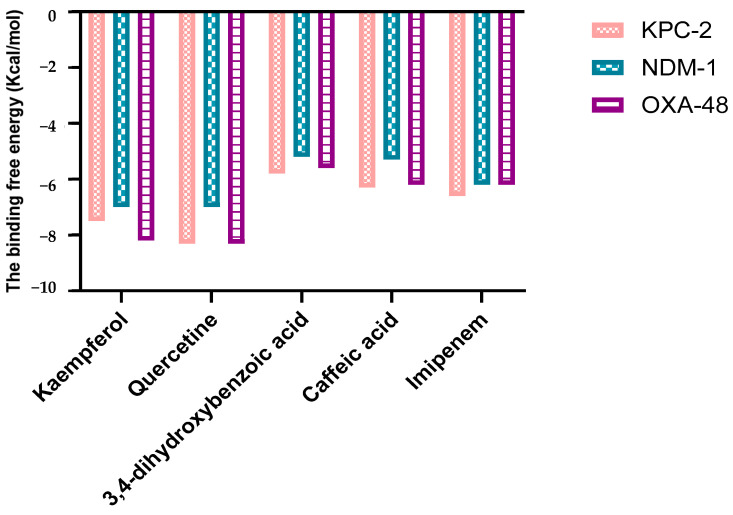
The estimated free binding energy (in kcal/mol) of quercetin, kaempferol, caffeic acid, 3,4-dihydroxybenzoic acid, and imipenem to the three carbapenemases.

A molecular docking study was carried out to investigate the binding mode of each polyphenol with three targets involved in carbapenem resistance (KPC-2, NDM-1, and OXA-48-like). The four compounds were docked into the active site of each receptor. The results (Figure 2) revealed that quercetin achieved a binding energy equal to −8 Kcal/mol against KPC-2 and OXA-48-like, followed by kaempferol with a binding affinity equal to −8, −7.5, and −6.5 Kcal/mol for OXA-48, KPC-2, and NDM-1, respectively. However, 3,4-dihydroxybenzoic acid showed affinity values comparable to the reference (imipenem).

**Figure 3:**
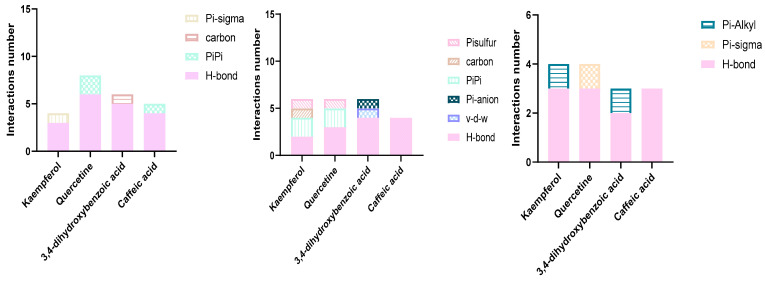
Types and number of molecular docking interactions between amino acid residues and the four polyphenols.

**Figure 4:**
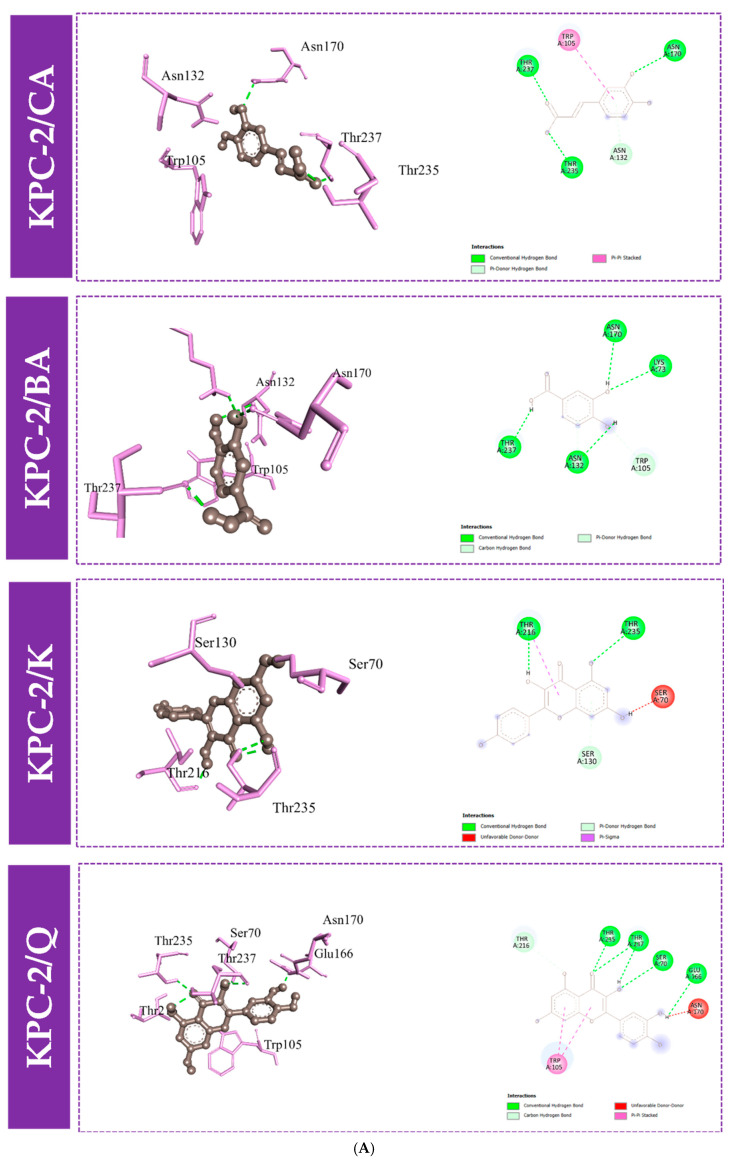

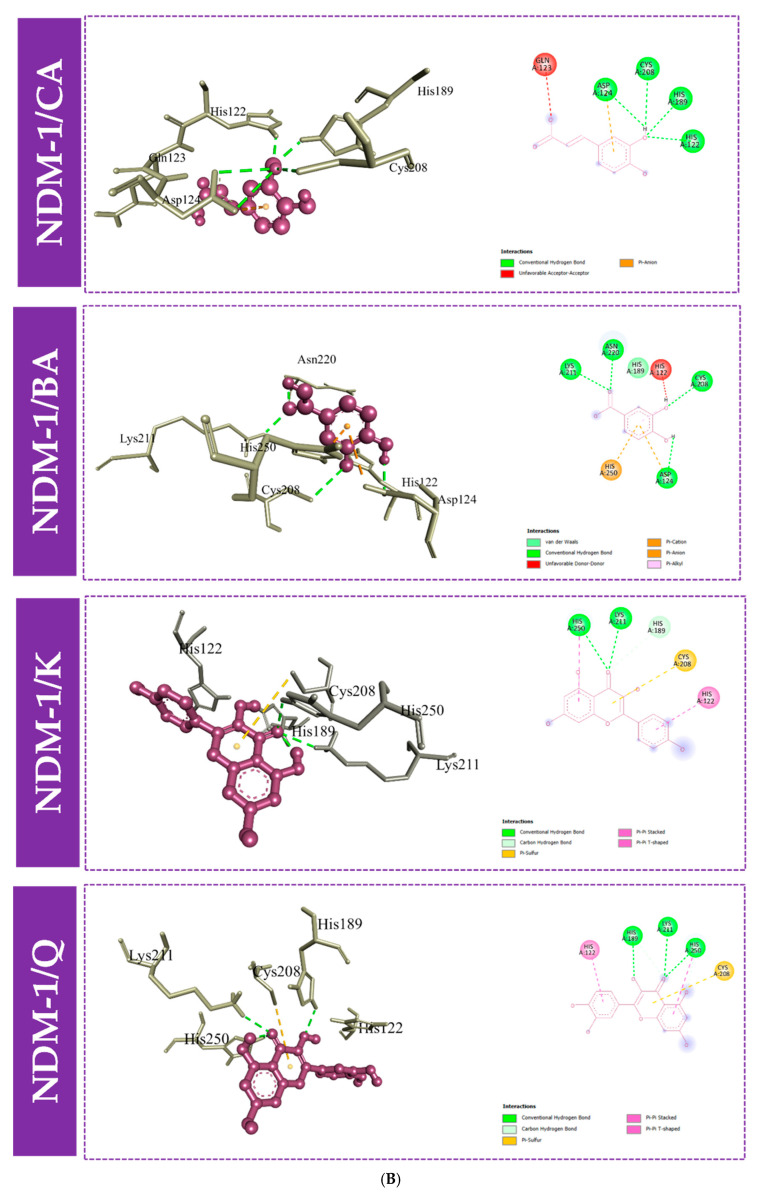

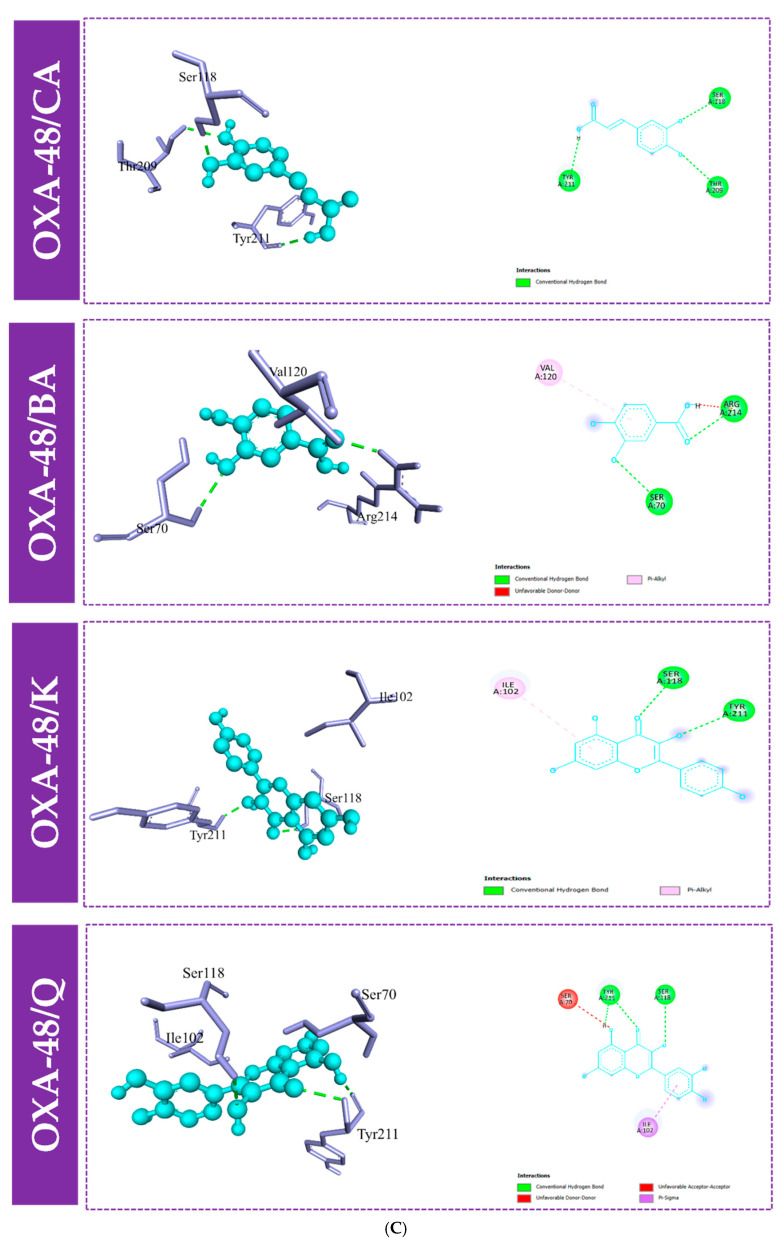
Molecular docking interactions between the amino acid residues of KPC-2 (**A**), NDM-1 (**B**), and OXA-48-like (**C**) and the four compounds, with a 3D and 2D view. CA: caffeic acid; BA: 3,4-dihydroxybenzoic acid; K: kaempferol; Q: quercetin.

Furthermore, to gain an understanding of the binding mode of the four compounds in the active site of the proteins KPC-2, OXA-48, and NDM-1, we performed 3D and 2D interaction analysis of the docked complexes using the Discovery Studio (DS) visualizer, as demonstrated in Figure 3 and Figure 4. Results revealed that different types of intermolecular interactions were formed between the ligands and the three targets. Quercetin binds to KPC-2 with Thr235, Thr237, Ser70, and Glu166 via hydrogen bonds and to Trp105 by Pi-Pi stacked interactions. Quercetin also interacted with His250, Lys211, and His189 with hydrogen bonds, established Pi-Pi stacked interactions with His122, and established Pi-sulfur interactions with Cys208 with NDM-1. Moreover, quercetin presented three interactions with Tyr211 and Ser118 via hydrogen bonds and with Ile102 by Pi-sigma interaction (Figure 3 and Table S1).

#### 2.1.2. Molecular Dynamics Simulations

Molecular dynamics (MD) simulations were performed to better understand the dynamic behavior and the binding mechanism of these polyphenols in a complex with the pocket of each receptor. To evaluate the stability of each complex for a period of 100 ns, we considered the root mean square fluctuation (RMSF), radius of gyration (Rg), hydrogen bonds (Hbs), and root mean square deviation (RMSD). Six plots are generated for each receptor: one corresponds to the receptor only (negative control), a second one corresponds to the receptor with imipenem (positive control), and the other four plots show the dynamics generated between the tested receptor–ligands. These plots of results allowed us to compare the differences between all the simulated complexes, which made it possible for us to highlight the most stable ligand.

##### The Radius of Gyration Trajectories

**Figure 5:**
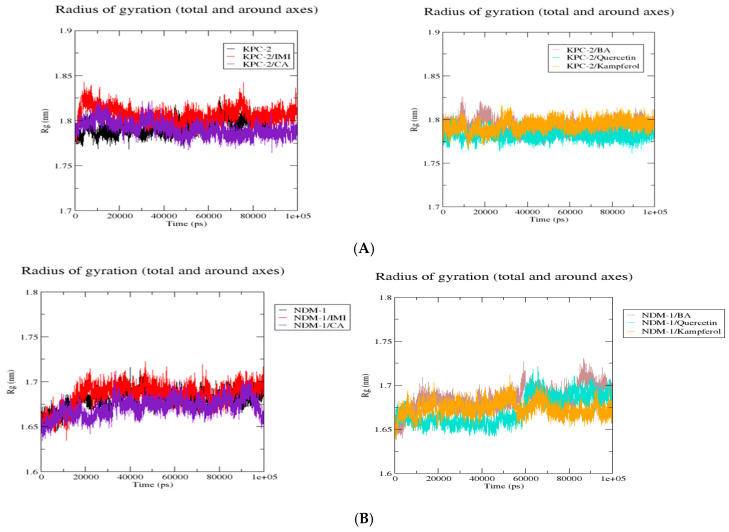

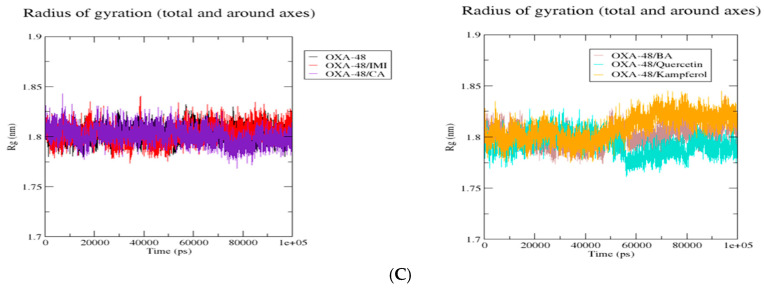
Comparative radius of gyration (Rg) values of KPC-2 (**A**), NDM-1 (**B**), and Oxa-48 (**C**) with the different ligands over 100 ns MD simulations. Rg (nm) is plotted as a function of time (ps). Black: protein alone; red: imipenem (IMI) complex; purple: caffeic acid (CA) complex; brown: 3,4-dihydroxybenzoic acid (BA) complex; cyan: quercetin complex; and orange: kaempferol complex. All systems exhibit stable Rg values, indicating overall structural stability throughout the simulations.

The superposition of Rg complexes plots of the KPC-2 protein (Figure 5) showed that the six complexes showed more or less the same behavior with a decrease in Rg values when the proteins were complexed onto the protein. This is the same case as complexes with NDM-1 and OXA-48. However, in the case of OXA-48, OXA-48/kaempferol showed an increase in Rg values after 50,000 ps.

##### Hydrogen Bond Number Analysis

**Figure 6:**
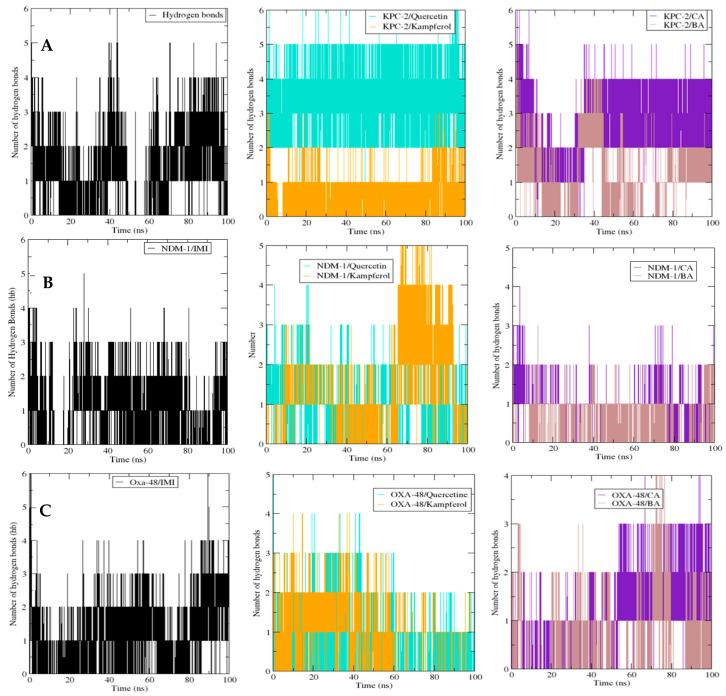
Comparative hydrogen bond number between the β-lactamases and their ligands for KPC-2 (**A**), NDM-1 (**B**), and Oxa-48 (**C**), and the different ligands over 100 ns MD simulations. The number of hydrogen bonds is plotted as a function of time (ps). Black: imipenem (IMI) complex; purple: caffeic acid (CA) complex; brown: 3,4-dihydroxybenzoic acid (BA) complex; cyan: quercetin complex; and orange: kaempferol complex. The stable hydrogen bond patterns suggest consistent interactions throughout the simulations.

The potential hydrogen bond (Hb) number measurement between active residues in protein targets and the chosen ligands during 100 ns was also plotted, analyzed, and compared to the interaction between imipenem (IMI) and protein targets (Figure 6). Then, hydrogen bonding in KPC-2 with the five ligands was estimated. The maximum number of HB (around five) was obtained with imipenem, caffeic acid, and kaempferol, followed by 3,4-dihydroxybenzoic acid and quercetin, forming four HB. In the case of NDM-1, it must be remarked that the maximum number of Hb incorporated was observed to be six for quercetin, four for imipenem, and three Hb for caffeic acid and kaempferol. In the case of the OXA-48 protein, all the ligands gained more than three Hb, but the ones with the highest number (four) were imipenem and 3,4-dihydroxybenzoic acid.

##### RMSD

**Figure 7:**
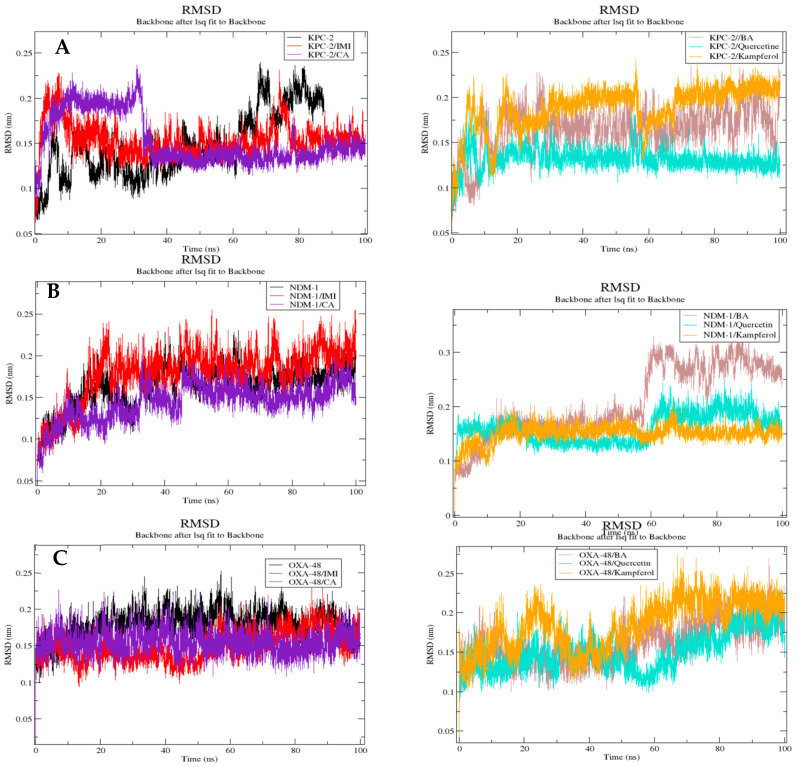
Comparative RMSD of trajectory plots after least squares fitting to the backbone atoms of each protein: KPC-2 (**A**), NDM-1 (**B**), and Oxa-48 (**C**) complexed with different ligands over 100 ns MD simulations. RMSD (nm) is plotted as a function of time (ps). Black: protein alone; red: imipenem (IMI) complex; purple: caffeic acid (CA) complex; brown: 3,4-dihydroxybenzoic acid (BA) complex; cyan: quercetin complex; and orange: kaempferol complex. All systems show stable RMSD values, indicating structural convergence during the simulations.

The RMSD results are presented in Figure 7. The RMSD fluctuations in the six complexes for the KPC-2 receptor ranged between 0.07 and 0.023 nm, 0.09 and 0.23 nm, 0.1 and 0.23 nm, 0.09 and 0.17 nm, 0.9 and 0.22 nm, respectively, for KPC-2 only, KPC-2/imipenem, KPC-2/caffeic acid, KPC-2/3,4-dihydroxybenzoic acid, KPC-2/quercetin, and KPC-2/kaempferol. The NDM-1 receptor ranged between 0.08 nm and 0.23 nm, 0.07 and 0.24 nm, 0.07 and 0.18 nm, 0.07 and 0.31 nm, 0.07 and 0.21 nm, and 0.07 and 0.2 nm for NDM-1 protein only, compounds imipenem, caffeic acid, 3,4-dihydroxybenzoic acid, quercetin, and kaempferol with the NDM-1, respectively, with 0.11 and 0.26 nm, 0.09 and 0.23 nm, 0.12 and 0.21 nm, 0.12 and 0.2, 0.1 and 0.19 nm, 0.11 and 0.26 nm, for OXA-48-like only, compounds imipenem, caffeic acid, 3,4-dihydroxybenzoic acid, quercetin, and kaempferol with the OXA-48-like, respectively.

##### RMSF Profiles of KPC-2 and NDM-1, OXA-48-like Proteins for Each Polyphenol

**Figure 8:**
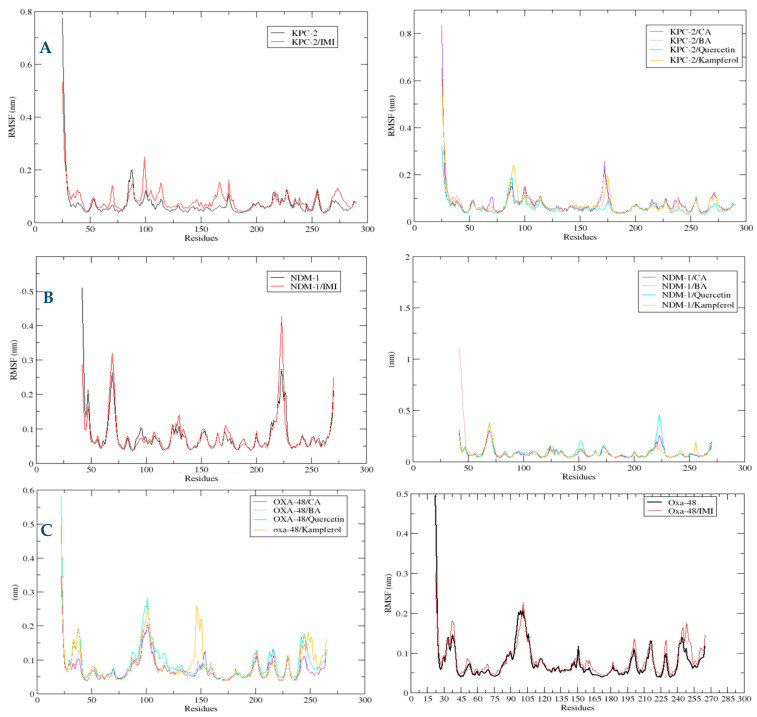
Comparative root mean square fluctuation (RMSF) of atoms for KPC-2 (**A**), NDM-1 (**B**), and OXA-48 (**C**) in the complex with different ligands over 100 ns MD simulations. RMSF (nm) is plotted as a function of residue number. Black: protein alone; red: imipenem (IMI) complex; purple: caffeic acid (CA) complex; brown: 3,4-dihydroxybenzoic acid (BA) complex; cyan: quercetin complex; orange: kaempferol complex. Residues showing higher RMSF values indicate regions of increased flexibility upon ligand binding.

To promote an understanding of the flexibility of protein structure, determine protein stability, and evaluate the average fluctuation per protein backbone residue, RMSF analysis was used (Figure 8). Concerning the KPC-2 plots, the amino acids which were more flexible in the KPC-2 were SER53, ALA88, GLY175, and GLY255. In the NDM-1, the protein structures of the six systems and the four polyphenols share similar RMSF distributions in the target protein. It showed that there are ten main flexible protein intervals corresponding to bands of residues Gly47, Gly69, ASP124, Asp130, and Asp223. Regarding the RMSF of OXA-48-like complexes, the most flexible residues in the systems are Asp101, Gly151, Gly201, Pro217, Asp229, and Asp245.

#### 2.1.3. Drug Likeness, ADMET Screening, and Toxicity Prediction

After molecular docking studies, the four compounds were subjected to physicochemical profiling using SwissADME. In the first step of in silico prediction, drug-like properties were assessed for Lipinski’s rule with drug-likeliness and physicochemical properties (number of rotatable bonds, number of hydrogen bond donors, and number of hydrogen bond acceptors) as shown in Table 1. The results obtained showed that all the evaluated molecules pass Lipinski’s rule with good solubility in water. Concerning toxicity prediction, it was evaluated with Protox II, and it revealed that kaempferol and caffeic acid belong to class 5, with an estimated LD_50_ exceeding 2000 mg/kg, respectively. For comparison, 3,4-dihydroxybenzoic acid in class 4 has an estimated LD_50_ between 300 and 2000 mg/kg, while quercetin in class 3 has LD_50_ values between 50 and 300 mg/kg, as shown in Table 2.

### 2.2. In Vitro Validation

#### 2.2.1. Effects of the Four Polyphenols on the Bacterial Cell Viability with AlamarBlue Assay

The AlamarBlue method was used to investigate the antibacterial activity of polyphenols. MICs of the four polyphenols against the six strains were determined, and the values are displayed in Figure 7 and Table 3. The results show very similar MICs for all the tests, with values of 12.5 or 25 mg/L depending on the polyphenol–strain combination.

**Figure 9:**
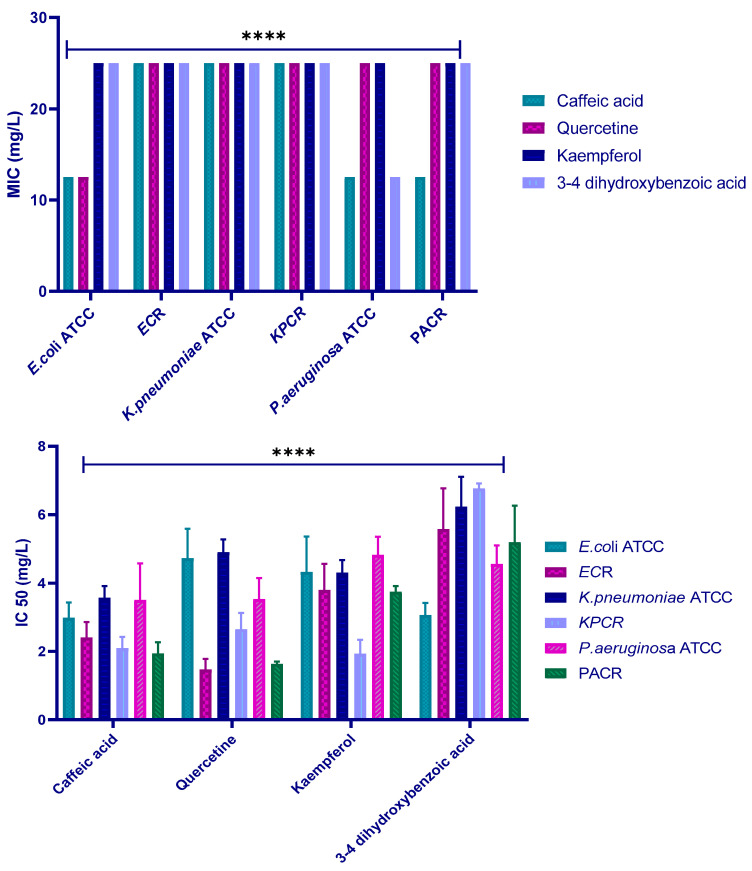
Minimum inhibitory concentration (MIC) and IC_50_ values (mg/L) of the four polyphenols against the six tested strains. (ECR: *E. coli* carbapenem-resistant; KPR: *K. pneumoniae* carbapenem-resistant; PAR: *P. aeruginosa* carbapenem-resistant). Data represent mean ± SD of three independent experiments (*n* = 3). **** indicates a statistically significant difference with *p* < 0.0001.

The AlamarBlue method was carried out to determine the IC50 value (Figure 9). The results revealed that among the tested polyphenols, caffeic acid exhibits the lowest IC50 value against *E. coli* ATCC25922, *K. pneumoniae* ATCC13883, and *P. aeruginosa* ATCC27853, with IC50 values of 2.98 mg/L, 3.56 mg/L, and 3.50 mg/L, respectively. Quercetin was the most effective against *E. coli* R and *P. aeruginosa* R with IC50 values of 1.46 mg/L and1.63 mg/L, respectively. In the case of *K. pneumoniae* R, kaempferol exhibited an IC50 of 1.92 mg/L. These data suggest that all the tested polyphenols have good inhibitory activity against the tested strains.

##### Minimum Bactericidal Concentration (MBC) Determination

**Figure 10:**
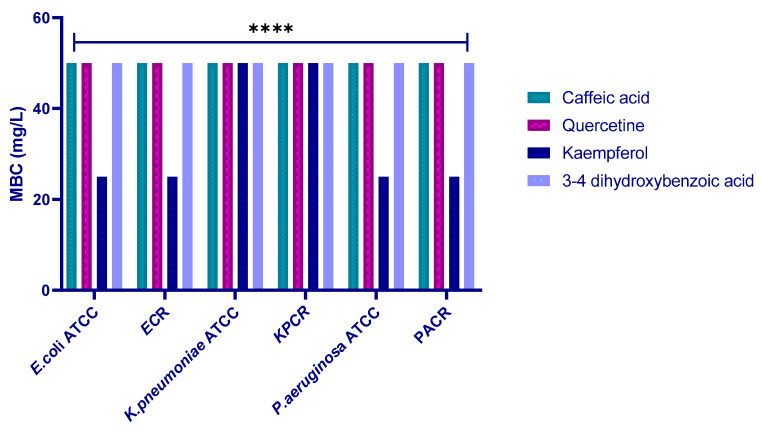
MBC values (mg/L) of the four polyphenols against the six tested strains (ECR: *E. coli* carbapenem-resistant; KPR: *K. pneumoniae* carbapenem-resistant; PAR: *P. aeruginosa* carbapenem-resistant). **** indicates a statistically significant difference with *p* < 0.0001.

To elucidate the bactericidal potential of the four tested molecules, the MBC was assessed against the included strains. The results revealed MBC values were 50 mg/L for all polyphenols against all the tested strains, except for kaempferol against most of them, which was 25 mg/L (Figure 10).

##### Checkerboard Assay

To determine the potential synergistic effects of the four polyphenols with cefotaxime (as a β-lactam) against the three carbapenem-resistant bacteria, checkerboard analysis was used (Figure S1, Supplementary Materials). Additionally, the effectiveness of these combinations was analyzed using the fractional inhibitory concentration index, which evaluates the interaction between cefotaxime and the four agents. Notably, the combination of cefotaxime and polyphenol revealed a synergistic action against both *E. coli* and *P. aeruginosa* when the polyphenol was quercetin, and against *P. aeruginosa* in association with kaempferol. The association of cefotaxime with caffeic acid and 3,4-dihydroxybenzoic acid turned out to be additive for the three tested strains, as presented in Table 4. None of the combinations demonstrated an antagonistic effect.

##### Polyphenols Inhibited β-Lactamase Activity

**Figure 11:**
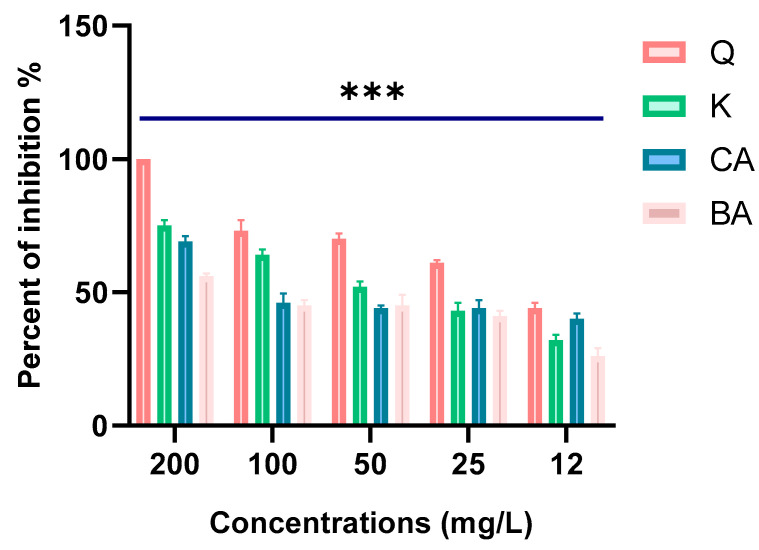
Percentage of β-lactamase activities inhibited by the four polyphenols. (Q: quercetin; K: kaempferol; CA: caffeic acid; BA: 3,4-dihydroxybenzoic acid). *** indicates a statistically significant difference with *p* < 0.001.

The four polyphenols included in this study markedly inhibited the hydrolytic potential of β-lactamase. The percentages among the tested compounds (as shown in Figure 11) were 100%, 75%, 69%, and 56% for quercetin, kaempferol, caffeic acid, and 3,4-dihydroxybenzoic acid, respectively, at 200 mg/L.

## 3. Discussion

The supremacy of carbapenem-resistant Gram-negative bacteria (CR-GNB) presents a worldwide healthcare emergency [3]. Previous studies have demonstrated that infections with MDR bacteria, including CR-GNB, are related to high mortality rates [7]. Furthermore, carbapenem resistance is predominantly caused by the production of carbapenemases, a type of β-lactamases that mainly exists in bacteria [8]. As far as we know, numerous molecular docking studies have explored the action of natural products against β-lactamases. Polyphenols—secondary metabolites produced by plants, animals, and microorganisms—are generally considered safe and have demonstrated various biological activities, including antibacterial effects [9]. Several natural constituents, such as epicatechin, epigallocatechin gallate, and tannic acid, have shown a meaningful inhibitory action on β-lactamases, as evidenced by both in vitro and in silico analysis [2]. This study aimed to evaluate the inhibitory activity of β-lactamases by polyphenols, both in silico and in vitro, and to restore cefotaxime as an effective β-lactam antibiotic. In the search for molecules that could help restore the effectiveness of β-lactam antibiotics, the phenolic compounds quercetin, kaempferol, caffeic acid, and 3,4-dihydroxybenzoic acid were tested in this study to better understand their modes of action on the neutralization of carbapenemases.

The polyphenolic compounds in this study were assessed in silico to prove their inhibitory actions on carbapenemase receptors KPC-2, NDM-1, and OXA-48. Molecular docking was conducted to show the interactions of the polyphenols with the target and to understand the binding mode. The results of the docked complexes (Figure 1) revealed that quercetin and kaempferol present the strongest binding affinities towards the targets, suggesting their potential as effective inhibitors. In addition, the analysis of the interaction profiles demonstrated that the predominant interaction of these compounds involved hydrogen bonds. Subsequently, molecular dynamics simulations were conducted to elucidate the stability of the complexes and the dynamic behavior over 100 ns. These calculations were performed using various parameters, RMSF, Rg, Hb, and RMSD, as quantitative indicators, to evaluate the stability of the complexes using the scanning of the motion of protein chain atoms in the presence of ligands [10]. The analysis of the four parameters for dynamics simulations suggested that quercetin and caffeic acid were the most consistent with the enzymes throughout the simulation period, compared to imipenem (the control). The imipenem/KPC-2 complex appears to be the most flexible system, followed by kaempferol/KPC-2, 3,4-dihydroxybenzoic acid/KPC-2. Moreover, quercetin and caffeic acid complexes with KPC-2 have low flexibility. Notably, less conformational flexibility was obtained with the system polyphenols/NDM-1. Generally, it should be noted that the less flexible and hence most stable complex is caffeic acid/OXA-48 (Table S2).

Before examining the in vitro activity of the studied phytochemicals, drug-likeliness and physicochemical properties of the molecules were predicted employing the SwissADME online server at http://www.swissadme.ch (accessed on 24 April 2025) [11]. No negative results were detected in the ADMET analysis of the compounds studied. All polyphenols were in line with Lipinski’s rule and none of the polyphenols evaluated revealed acute toxicity, indicating their potential as drug-like compounds. Thereafter, we examined the topological polar surface area (TPSA) as a physicochemical descriptor of polarity, which should be between 20 and 130(Å^2^) for optimal drug absorption and permeability. Results collected in Table 1 and Table 2 show that all ligands had values falling within the above-mentioned range, except for quercetin. These findings highlight polyphenols as promising candidates for further investigation, as they exhibited both strong binding affinity and enhanced stability in the enzyme–ligand complex.

Subsequently, to further validate the in silico results, we performed in vitro experiments. The four polyphenols included in this study showed good antibacterial activity against the target strains, and we identified quercetin, caffeic acid, and kaempferol as the most potent antibacterial agents (Figure 7). The antibacterial evaluation revealed that the tested polyphenols exhibited bactericidal activity against the studied bacterial strains, as indicated by MBC/MIC ratios ≤ 4, with MIC values ranging from 12.5 to 25 mg/L. These findings suggest that the inhibitory action of the compounds is both concentration- and strain-dependent. The combination assays with cefotaxime demonstrated synergistic or additive effects, supporting the hypothesis that polyphenols can enhance β-lactam efficacy by interfering with resistance mechanisms such as β-lactamase activity. Following the evaluation of the antibacterial and combination effects of the polyphenols, the effect of quercetin was further analyzed with consistent antibacterial effects and notable synergy with cefotaxime against carbapenem-resistant strains. The current findings support the idea that quercetin enhances antibiotic uptake and may interfere with β-lactamase-mediated hydrolysis. Mohammed et al. recently reviewed the bioactivities of quercetin, including the antibacterial activity against different bacterial strains, such as *P. aeruginosa* and *E. coli* [12]. Similarly, Zhang et al. demonstrated that polyphenols such as quercetin efficiently reinstated the antibacterial effectiveness of imipenem or piperacillin on *E. coli-*producing OXA-48, with a subsequent two- to eight-fold diminution of MIC [13]. Moreover, quercetin alone killed up to 99.95% of bacteria within 4–6 h of dosing, and it decreased the MIC value (64–1024 μg/mL) of meropenem [14]. Additionally, it established a synergistic effect with meropenem on 89.25% of carbapenem-resistant bacteria and exhibited bactericidal action by interrupting the cell wall and membrane stability, as well as modifying cellular morphology [14]. Furthermore, the combination of quercetin–ceftriaxone showed a synergistic effect on *P. aeruginosa* strains, which can also increase the efficacy of the antibiotic used in carbapenem-resistant *E. coli* [6,15]. In another study, quercetin alone (16 μg/mL) had no antibacterial activity, signaling that it did not affect *A. baumannii* growth. In contrast, adding 16 μg/mL of quercetin decreased the MIC of meropenem by four times by affecting the overexpression of OXA-98. These results suggested that quercetin can restore the in vitro effect of meropenem against OXA-98-producing *A. baumannii* [16].

Kaempferol also exhibited antibacterial activity and synergistic effects with cefotaxime. Similarly to the literature reports, kaempferol likely acts by inhibiting bacterial DNA gyrase and leading to cell membrane damage [17]. Although some studies have reported that kaempferol has no or low antibacterial activity, synergistic effects have been observed after its association of with colistin [18]. Indeed, our results confirm that kaempferol improves bacterial inhibition of β-lactams, when applied together, emphasizing its value as a combination adjuvant rather than a standalone antimicrobial.

In addition to the significant effect shown in our study by caffeic acid against the included strains, Araújo et al. (2019) conclude that caffeic acid has various biological activities, including antibacterial, and its derivatives are promising candidates for treating bacterial pathologies, especially against *E. coli* [19]. These findings are in line with those reported by other studies, which proved that caffeic acid supplementation inhibits the growth of *E. coli* and other Gram-negative bacteria [20,21].

In addition, our results regarding the moderate effect of benzoic acid were supported by other researchers. Previous studies showed that benzoic acid and its derivatives inhibit *K. pneumoniae’*s growth [22], and that its derivate, 3,4-dihydroxybenzoic acid, is the strongest inhibitory molecule among this group [23]. Another study showed that metabolites, such as 2,3-dihydroxybenzoic acid, significantly enhance the effect of tigecycline in the treatment of infections caused by *E. coli* and *K. pneumoniae* isolates harboring genes that encode carbapenemases or ESBLs [24]. This enhanced activity of hydroxybenzoic acids may be related to hydroxyl substitutions, which can raise the lipophilicity of the phenolic acid, thereby allowing a better interaction with the bacteria and improving antibacterial action [25].

As we had expected based on previous findings, all the compounds studied in this work displayed a very good inhibition of β-lactamases. Markedly, quercetin demonstrated potent inhibitory activity against β-lactamase, exhibiting complete inhibition (100%), thereby effectively suppressing its hydrolytic potential. Accordingly, the potent bactericidal effect of quercetin may be linked to the alteration in *bla*_VIM_ [26]. Pal et al. (2019) also demonstrated that quercetin inhibited the *bla*_NDM_ gene, which encodes a metallo-β-lactamase involved in carbapenem resistance [14]. In addition, kaempferol with ceftiofur exhibits synergistic bactericidal and antibacterial activities on *E. coli-*producing ESBLs by affecting the action of β-lactamases and inhibiting the hydrolytic activity of *bla_CTX-M-27_* protein, offering new guidance for our future research [27].

This research provides significant insights into the inhibitory potential of natural polyphenols against β-lactamase-producing bacteria by combining in silico and in vitro methods. Building on these promising findings, future research should focus on further characterizing the underlying mechanisms through enzyme kinetics and structure–activity relationship studies. In vivo studies would also be valuable in confirming the biological relevance of these compounds under physiological conditions. Extending these analyses to more classes of antibiotics could help define new synergistic combinations and strengthen the therapeutic prospects of polyphenols as adjunctive agents in the treatment of multidrug-resistant infections.

## 4. Materials and Methods

### 4.1. Exploring Computational Methods and Predictions

#### 4.1.1. Molecular Docking of Carbapenemase Inhibitors

##### Receptor Preparation, Active Site Prediction, and Grid Generation

The 3D crystal structures of protein receptors taken in this study, KPC-2, NDM-1, and OXA-48-Like, were retrieved in PDB format from the RCSB Protein Data Bank (https://www.rcsb.org/, accessed on 19 September 2024). The PDB IDs, namely 7E9A (for KPC-2), 5YPL (for NDM-1), and 6PK0 (for OXA-48-Like), were chosen based on their high crystallographic resolution (2.25 Å, 1.8 Å, 1.75 Å, respectively) and the presence of co-crystallized β-lactamase inhibitors, which ensured accurate active-site definition and reliable docking validation. We used the PDBsum database (http://www.ebi.ac.uk/thornton-srv/databases/pdbsum/, accessed on 19 September 2024) for mapping active-site amino acid residues that bind to the imipenem (IMI) to target protein sequences of interest. All water molecules, ions, and the bound ligand were deleted from the protein receptors of interest before docking by PyMol software version 4.6. Its crystal structures were used for in silico studies using MGTools (version 1.5.7) of AutoDock Vina software version 1.1.2. The PDB files were energy minimized, the non-essential water molecules were added, and the polar hydrogen atoms of the receptors were merged. Furthermore, the MGTools Autodock Vina was also used to specify the grid box. We specified the binding site with the amino acid residues that will be targeted in the molecular docking process. After that, the structure of the proteins was saved in PDBQT format.

The receptor grid box was set at the following coordinates: for KPC-2, the center to X = 95.787 Å, Y = 6.548 Å, Z = 217.641 Å, and the lattice size to 18 Å × 22 Å × 20 Å. For NDM-1, the center to X = −40.694 Å, Y = 77.397 Å, Z = 97.51 Å, and the lattice size to 18 Å × 20 Å × 20 Å. For OXA-48-like and NDM-1, the center to X = 11.732 Å, Y = −16.522 Å, Z = 37.131 Å, and the lattice size to 20 Å × 16 Å × 22 Å.

##### Compound Preparation

The chemical compounds (quercetin, kaempferol, caffeic acid, and 3,4-dihydroxybenzoic acid) are used in molecular docking to predict their potential as inhibitors of carbapenemases. The chemical structures of the ligands for docking were retrieved from the PubChem database (https://pubchem.ncbi.nlm.nih.gov/, accessed on 19 September 2024), and their 3D structures were downloaded in SDF (Structure Data File) format and then converted into PDB (Protein Data Bank) format using PyMOL and AutoDock Tools were used to translate the ligand into PDBQT format.

##### Molecular Docking Simulations

Molecular docking of the four constituents targeting proteins of interest was performed by Autodock Vina Version 1.1.2. All the PDBQT structures of ligands were docked in the binding sites of the proteins using the standard parameters of Autodock Vina. The docking scores were calculated as the predicted binding free energies (kcal/mol). Biovia Discovery Studio Visualizer 2021 was used to determine hydrogen bonds and non-bonding interactions between the ligand and receptor, and to search for complex structures that interacted with amino acids and docked poses on both 3D and 2D diagrams. The reliability of the docking protocol was confirmed through redocking of the native inhibitor into each enzyme’s active site, followed by comparison of its binding conformation and key interactions with those of the reference compound, imipenem.

#### 4.1.2. Molecular Dynamics Simulations Using Gromacs

Inhibitors and protein complexes were simulated by molecular dynamics (MD). MD was carried out to investigate stability and to understand the dynamic behavior and binding mechanism of the selected compounds obtained by AutoDock Vina in complexes with the three targets, using the Gromacs 2021.3 software package, compared to the MD of free proteins [28].

All the computational preparations and the MD production steps of the selected complex were carried out in the High-Performance Computing cluster MARWAN (HPC-MARWAN, GPU) at the National Center for Scientific and Technical Research (CNRST), Rabat, Morocco (https://hpc.marwan.ma/). The root mean square fluctuation (RMSF), the radius of gyration (Rg), the root mean square deviation (RMSD), the energy, and the hydrogen bonds (hbs) were analyzed throughout their trajectory plots. The trajectory plotting was carried out by the Qtgrace software v0.2.7 [29]. Each molecular dynamics simulation was performed once for 100 ns due to computational limitations. Therefore, no statistical averaging over multiple trajectories was applied.

#### 4.1.3. Drug Likeness, ADMET, and Toxicity Prediction

It is commonly known that the ADMET characteristics of drugs should be considered as early as possible to reduce failure rates in the clinical phase of drug development [30]. Toxicity prediction, drug likeness activity, and ADMET screening of the polyphenols included were carried out. Lipinski’s rule of five [30] was employed to predict drug-likeness properties like absorption, distribution, metabolism, excretion, and toxicity; the rule estimates that there is likely to be poor absorption or permeation when a component possesses a certain number of hydrogen donors (should not be more than 5) and hydrogen acceptors (the number of should not be more than 10); partition coefficient log P should be less than 5 and molecular weight or mass should not be more than 500 Daltons; it was conducted through the online tool SwissADME (http://www.swissadme.ch/). 

Therefore, we used the Pro-Tox-II web server (https://tox-new.charite.de/protox_II/index.php?site=compound_input, accessed on 13 April 2025) to estimate the toxicity class of the natural compounds and their LD50 value.

### 4.2. Experimental Confirmation and Biological Evaluation

#### 4.2.1. Polyphenols and Antibiotic

The phytochemicals used in this study, kaempferol (K) and 3,4-dihydroxybenzoic acid (BA), were purchased from Zwijndrecht, Belgium. Quercetin (Q) and caffeic acid (CA) were purchased from Steinheim, Germany. All the tested compounds were dissolved in propylene glycol (Scharlab, Barcelona, Spain) to prepare the respective stock solutions. Cefotaxime (Ctx) was from Sigma-Aldrich (Burlington, MA, USA).

#### 4.2.2. Bacterial Strains

The current study included six Gram-negative bacteria. Three reference strains, *P. aeruginosa* ATCC27853, *K. pneumoniae* ATCC13883, and *E. coli* ATCC25922, used for quality control, were obtained from the American Type Culture Collection (Manassas, VA, USA). Three clinical isolates (*K. pneumoniae* R, *E. coli* R, *P. aeruginosa* R) were isolated from urinary tract infections. These isolates were previously identified as carbapenemase-producing strains [31] and were stored in glycerol. Glycerol stocks were streaked on nutritive agar to obtain fresh cultures to be used for all further studies.

#### 4.2.3. Assessment of the Antimicrobial Activity Using the AlamarBlue Assay

The minimum inhibitory concentration (MIC) of all compounds against the above mentioned strains was determined using the broth microdilution guidelines of the Clinical and Laboratory Standards Institute (CLSI) [32], followed by determination of metabolic activity using the AlamarBlue method with fluorometric detection. Cells from an overnight bacterial culture on LB plates were resuspended in 2× cation-adjusted Müller Hinton II broth (CAMHB) to a final concentration of 5 × 10^5^ colony-forming units (CFUs)·mL^−1^. Serial dilutions of the four compounds (50, 25, 12.5, 6.25, 3.12 mg/L) in sterile phosphate-buffer saline (PBS) were prepared in sterile 96-well microplates, and the volume of each well was adjusted to 100 µL. A total of 100 µL of freshly prepared bacterial inoculum was introduced into each well to obtain a final total volume of 200 µL per well. Then the microplates were incubated for 18–24 h at 37 °C.

After incubation, the AlamarBlue method was utilized to assess the effect of the four compounds on the viability of the bacterial strains [33]. A total of 20 µL of resazurin solution (ThermoFisher Scientific, Waltham, MA, USA) was added to each well, and the mixture was incubated away from light until a colorimetric change was observed. Resazurin reduction into resorufin was measured using the FLUOstar microplate reader at an excitation wavelength of 544 nm and an emission wavelength of 590 nm. As a control, resazurin in PBS without bacterial culture was included.

For data on IC50, polyphenols’ concentration was needed for 50% inhibition of bacterial growth and it was calculated by GraphPad Prism 9.0.

#### 4.2.4. Minimum Bactericidal Concentration (MBC Determination)

To determine the minimum bactericidal concentrations (MBCs) of the four polyphenols on the target strains, samples from the wells that did not exhibit any metabolism in the MIC assay were spread on LB plates and then incubated at 37 °C for 24 h. After incubation, the lowest concentration of the polyphenol that effectively killed all the bacterial population, and hence produced no colony on LB agar, was considered the MBC [34].

#### 4.2.5. Checkerboard Assay

The synergistic effects between the four compounds and cefotaxime were tested against the clinical strains by performing a checkerboard microdilution method as described previously [35]. The bacterial strains used in this assay were classified as resistant to cefotaxime (CTX), as the MIC is equal to or higher than 2 mg/L. Each compound and cefotaxime were serially diluted, starting with concentrations of 50 mg/L and 0.5 mg/mL, respectively. After dilution in a 96-well plate, 100 μL inoculum of 10^6^ CFU/mL of bacteria was added. Afterwards, the 96-well plate was incubated at 37 °C for 18–24 h.

The synergism was verified by calculating the fractional inhibitory concentration index (FICI). FICI was calculated according to the following formulas: FIC for polyphenol = MIC polyphenol in combination/MIC of polyphenol alone; FIC for cefotaxime = MIC of cefotaxime in combination/MIC of cefotaxime alone. Finally, FICI (FICI = FIC for polyphenol + FIC for cefotaxime) was calculated. Data were interpreted according to the following criteria: FICI ≤ 0.5 synergistic effect, 0.5 < FICI ≤ 4 additive effect, and FICI > 4 antagonistic effect [35].

#### 4.2.6. Determination of % of β-Lactamase Inhibition

The inhibitory effects of the four bioactive compounds (quercetin, kaempferol, caffeic acid, and 3,4-dihydroxybenzoic acid) on β-lactamases were assessed employing the nitrocefin hydrolysis assay, following the manufacturer’s instructions provided in the kit (AAT Bioquest’s Colorimetric Beta-lactamase Activity Assay Kit, Sunnyvale, CA, USA). The results were measured in 96-well plates at 37 °C using a microplate reader. The hydrolysis and inhibition percentages were calculated as described previously, employing the following formulae: % of inhibition = [(OD of treated − OD control)/OD of treated] × 100, where % of hydrolysis  =  100 − (% of inhibition) [4].

#### 4.2.7. Statistical Analysis

GraphPad Prism version 9.0 for Windows was used to calculate the values of IC50 of the AlamarBlue assay. Statistical analysis was performed using one-way ANOVA followed by Dunnett’s post hoc test.

## 5. Conclusions

To summarize, this study highlights the potential of selected polyphenols—quercetin, kaempferol, caffeic acid, and 3,4-dihydroxybenzoic acid—as effective inhibitors of β-lactamase. Equally important, combined in silico and in vitro approaches revealed that the four polyphenols exert excellent inhibitory action on β-lactamase and ability to enhance the activity of β-lactam antibiotics against carbapenem-resistant Gram-negative strains. This could significantly strengthen the pipeline in combination therapy. These findings provide valuable insights into the mechanism of polyphenol-mediated β-lactamase inhibition and support their further investigation as adjuncts in combination therapy against multidrug-resistant bacteria.

## Figures and Tables

**Table 1:** Physicochemical properties and drug-likeness prediction of the 18 top hits by ADMET and evaluation of drug-likeness rules using SwissADME.

Polyphenols	MW (g/mol)	LogP	H-Bond Acceptors	H-Bond Donors	Lipinski Rule	TPSA (Å^2^)	Solubility	Bioavailability Score
Kaempferol	286.24	2.28	6	4	Yes	111.13	Pass	0.55
Quercetin	302.24	1.99	7	5	Yes	131.36	Pass	0.55
Caffeic acid	180.16	1.09	4	3	Yes	77.76	Pass	0.56
3,4-Dihydroxybenzoic acid	154.12	0.80	4	3	Yes	77.76	Pass	0.56

**Table 2:** Toxicity prediction using ProTox software 3.0.

Polyphenols	LD50 mg/kg	Toxicity Class
Kaempferol	3919	5
Quercetin	159	3
Caffeic acid	2980	5
3,4-Dihydroxybenzoic acid	2000	4

**Table 3:** MIC values (mg/L) of the four polyphenols against the tested strains.

	Caffeic Acid	Quercetin	Kaempferol	3,4-Dihydroxybenzoic Acid
*E. coli* ATCC25922	12.5	12.5	25	25
*E. coli* R	25	25	25	25
*K. pneumoniae ATCC13883*	25	25	25	25
*K. pneumoniae* R	25	25	25	25
*P. aeruginos*a ATCC27853	12.5	25	25	12.5
*P. aeruginosa* R	12.5	25	25	25

**Table 4:** The fractional inhibitory concentration index (FICI) values and corresponding effect classifications of the combinations of cefotaxime with the four polyphenols. (Q: quercetin; K: kaempferol; CA: caffeic acid; BA: 3,4-dihydroxybenzoic acid; Ctx: Cefotaxime; ECR: *E. coli* carbapenem-resistant; KPR: *K. pneumoniae* carbapenem-resistant; PAR: *P. aeruginosa* carbapenem-resistant).

	Ctx/Q		Ctx/CA		Ctx/K		Ctx/BA	
	FICI	Effect	FICI	Effect	FICI	Effect	FICI	Effect
KPR	0.5283	additive	0.7267	additive	1.0667	additive	0.7078	additive
PAR	0.2784	synergistic	0.8667	additive	0.2584	synergistic	0.78	additive
ECR	0.2351	synergistic	0.52	additive	0.52	additive	0.8578	additive

## Data Availability

The original contributions presented in this study are included in the article/Supplementary Materials. Further inquiries can be directed to the corresponding author.

## Supplementary Materials

The following supporting information can be downloaded at https://www.mdpi.com/article/10.3390/molecules30224416/s1, Figure S1: Effect of the combination of cefotaxime with the four polyphenols on *K. pneumonia*, *P. aeruginosa*, and *E. coli isolates*; Table S1: List the interacting amino acid residues involved in the ligand–protein interaction of the major; Table S2: Combined summary of molecular docking score, RMSD, Rg, RMSF, and hydrogen bond analyses for β-lactamase–polyphenol complexes over 100 ns MD simulations.

## References

[1] Bibi Z., Asghar I., Ashraf N.M., Zeb I., Rashid U., Hamid A., Ali M.K., Hatamleh A.A., Al-Dosary M.A., Ahmad R. (2023). Prediction of Phytochemicals for Their Potential to Inhibit New Delhi Metallo β-Lactamase (NDM-1). Pharmaceuticals.

[2] Abdel-Halim M.S., Askoura M., Mansour B., Yahya G., El-Ganiny A.M. (2022). In Vitro Activity of Celastrol in Combination with Thymol against Carbapenem-Resistant *Klebsiella pneumoniae* Isolates. J. Antibiot..

[3] Mourabiti F., Jouga F., Sakoui S., El Hosayny O., Zouheir Y., Soukri A., El Khalfi B. (2025). Mechanisms, Therapeutic Strategies, and Emerging Therapeutic Alternatives for Carbapenem Resistance in Gram-Negative Bacteria. Arch. Microbiol..

[4] Guo Y., Liu H., Yang M., Ding R., Gao Y., Niu X., Deng X., Wang J., Feng H., Qiu J. (2024). Novel Metallo-β-Lactamases Inhibitors Restore the Susceptibility of Carbapenems to New Delhi Metallo-Lactamase-1 (NDM-1)-Harbouring Bacteria. Br. J. Pharmacol..

[5] Terbtothakun P., Nwabor O.F., Siriyong T., Voravuthikunchai S.P., Chusri S. (2021). Synergistic Antibacterial Effects of Meropenem in Combination with Aminoglycosides against Carbapenem-Resistant *Escherichia coli* Harboring BlaNDM-1 and BlaNDM-5. Antibiotics.

[6] Aydemir Ö., Ormanoğlu G., Ayhancı T., Zengin M., Köroğlu M. (2023). Investigation of in Vitro Efficacy of Quercetin-Meropenem Combination in Carbapenemase-Producing *Klebsiella pneumoniae* Isolates. J. Infect. Dev. Ctries..

[7] Kuloglu T.O., Unuvar G.K., Cevahir F., Kilic A.U., Alp E. (2024). Risk Factors and Mortality Rates of Carbapenem-Resistant Gram-Negative Bacterial Infections in Intensive Care Units. J. Intensive Med..

[8] Ma J., Song X., Li M., Yu Z., Cheng W., Yu Z., Zhang W., Zhang Y., Shen A., Sun H. (2023). Global Spread of Carbapenem-Resistant Enterobacteriaceae: Epidemiological Features, Resistance Mechanisms, Detection and Therapy. Microbiol. Res..

[9] Etminani F., Etminani A., Hasson S.O., Judi H.K., Akter S., Saki M. (2023). In Silico Study of Inhibition Effects of Phytocompounds from Four Medicinal Plants against the Staphylococcus Aureus β-Lactamase. Inform. Med. Unlocked.

[10] Tabti K., Abdessadak O., Sbai A., Maghat H., Bouachrine M., Lakhlifi T. (2023). Design and Development of Novel Spiro-Oxindoles as Potent Antiproliferative Agents Using Quantitative Structure Activity Based Monte Carlo Method, Docking Molecular, Molecular Dynamics, Free Energy Calculations, and Pharmacokinetics /Toxicity Studies. J. Mol. Struct..

[11] Daina A., Michielin O., Zoete V. (2017). SwissADME: A Free Web Tool to Evaluate Pharmacokinetics, Drug-Likeness and Medicinal Chemistry Friendliness of Small Molecules. Sci. Rep..

[12] Mohammed F.S., Sevindik M., Uysal İ., Sabik A.E. (2023). Quercetin: Derivatives, Biosynthesis, Biological Activity, Pharmacological and Therapeutic Effects. Prospect. Pharm. Sci..

[13] Zhang Y., Chen C., Cheng B., Gao L., Qin C., Zhang L., Zhang X., Wang J., Wan Y. (2022). Discovery of Quercetin and Its Analogs as Potent OXA-48 Beta-Lactamase Inhibitors. Front. Pharmacol..

[14] Pal A., Tripathi A. (2019). Quercetin Potentiates Meropenem Activity among Pathogenic Carbapenem-Resistant *Pseudomonas aeruginosa* and *Acinetobacter baumannii*. J. Appl. Microbiol..

[15] Vipin C., Saptami K., Fida F., Mujeeburahiman M., Rao S.S., Athmika, Arun A.B., Rekha P.D. (2020). Potential Synergistic Activity of Quercetin with Antibiotics against Multidrug-Resistant Clinical Strains of *Pseudomonas aeruginosa*. PLoS ONE.

[16] Yang Y., Song J., Gao Y., Li D., Li X., Liu Y. (2023). The Inhibition Mechanism of Quercetin Targeting β-Lactamases OXA-98 Based on Molecular Dynamics Simulation. Chem. Phys. Lett..

[17] Periferakis A., Periferakis K., Badarau I.A., Petran E.M., Popa D.C., Caruntu A., Costache R.S., Scheau C., Caruntu C., Costache D.O. (2022). Kaempferol: Antimicrobial Properties, Sources, Clinical, and Traditional Applications. Int. J. Mol. Sci..

[18] Zhou H., Xu M., Guo W., Yao Z., Du X., Chen L., Sun Y., Shi S., Cao J., Zhou T. (2022). The Antibacterial Activity of Kaempferol Combined with Colistin against Colistin-Resistant Gram-Negative Bacteria. Microbiol. Spectr..

[19] Araújo M.O., Freire Pessoa H.L., Lira A.B., Castillo Y.P., De Sousa D.P. (2019). Synthesis, Antibacterial Evaluation, and QSAR of Caffeic Acid Derivatives. J. Chem..

[20] Kauffmann A.C., Castro V.S. (2023). Phenolic Compounds in Bacterial Inactivation: A Perspective from Brazil. Antibiotics.

[21] Xu T., Zhu H., Liu R., Wu X., Chang G., Yang Y., Yang Z. (2022). The Protective Role of Caffeic Acid on Bovine Mammary Epithelial Cells and the Inhibition of Growth and Biofilm Formation of Gram-Negative Bacteria Isolated from Clinical Mastitis Milk. Front. Immunol..

[22] Rohatgi A., Gupta P. (2023). Benzoic Acid Derivatives as Potent Antibiofilm Agents against *Klebsiella pneumoniae* Biofilm. J. Biosci. Bioeng..

[23] Yuan J.J., Yan H.J., He J., Liu Y.Y. (2021). Antibacterial Activities of Polyphenols from Olive Leaves against *Klebsiella pneumoniae*. IOP Conf. Ser. Earth Environ. Sci..

[24] Farooq A., Drotleff B., Kroemer N., Han M.L., Li J., Decousser J.W., Schrey D., Buyck J., Grégoire N., Nordmann P. (2025). Evaluation of in Vitro Pharmacodynamic Drug Interactions of Ceftazidime-Avibactam with Tigecycline in ESBL- and Carbapenemase Producing *Escherichia coli* and *Klebsiella pneumoniae*. Int. J. Antimicrob. Agents.

[25] Forero-Doria O., Parra-Cid C., Venturini W., Espinoza C., Araya-Maturana R., Valenzuela-Riffo F., Saldias C., Leiva A., Duarte Y., Echeverría J. (2022). Novel N-Benzoylimidazolium Ionic Liquids Derived from Benzoic and Hydroxybenzoic Acids as Therapeutic Alternative against Biofilm-Forming Bacteria in Skin and Soft-Tissue Infections. Bioorg. Chem..

[26] Pal A., Tripathi A. (2020). Demonstration of Bactericidal and Synergistic Activity of Quercetin with Meropenem among Pathogenic Carbapenem Resistant *Escherichia coli* and *Klebsiella pneumoniae*. Microb. Pathog..

[27] Li P.C., Tong Y.C., Xiao X.L., Fan Y.P., Ma W.R., Liu Y.Q., Zhuang S., Qing S.Z., Zhang W.M. (2024). Kaempferol Restores the Susceptibility of ESBLs *Escherichia coli* to Ceftiofur. Front. Microbiol..

[28] Berendsen H.J.C., Van Der Spoel D., Van Drunen R. (1995). GROMACS: A Message-Passing Parallel Molecular Dynamics Implementation. Comput. Phys. Commun..

[29] Van Der Spoel D., Lindahl E., Hess B., Groenhof G., Mark A.E., Berendsen H.J.C. (2005). GROMACS: Fast, Flexible, and Free. J. Comput. Chem..

[30] Lipinski C.A., Dominy B.W., Feeney P.J. (1997). Drug Delivery Reviews Experimental and Computational Approaches to Estimate Solubility and Permeability in Drug Discovery and Development Settings. Adv. Drug Deliv. Rev..

[31] Mourabiti F., Jouga F., Mouzoun Y., Arslan S., Schena R., Zouheir Y., Soukri A., De Martino L., Nocera F.P., El Khalfi B. (2025). Phenotypic and genotypic characterization of carbapenem encoding genes among carbapenem-resistant Gram-negative bacteria isolated from North Casablanca, Morocco. Comp. Immunol. Microbiol. Infect. Dis..

[32] Lewis J.S., Amy Mathers F.J., April Bobenchik D.M., Alexandra Lynn Bryson D., Shelley Campeau D., Sharon Cullen D.K., Tanis Dingle R., German Esparza F., Humphries R.M., Thomas Kirn F.J. (2021). CLSI M100-Ed35 January 2025 Replaces CLSI M100-Ed34 Performance Standards for Antimicrobial Susceptibility Testing.

[33] Calvo L.G., Villarino R.A., Rama J.L.R., Abril A.G., de Miguel T. (2025). A Modification of the Resazurin Cell Viability Assay, Suitable for the Quantification of Lactic Acid Producing Bacteria. LWT.

[34] Mourabiti F., Derdak R., El Amrani A., Momen G., Timinouni M., Soukri A., El Khalfi B., Zouheir Y. (2024). The Antimicrobial Effectiveness of *Rosmarinus officinalis*, *Lavandula angustifolia*, and *Salvia officinalis* Essential Oils against *Klebsiella pneumoniae* and *Pseudomonas aeruginosa* in Vitro and in Silico. S. Afr. J. Bot..

[35] Güran M., Şanlıtürk G., Kerküklü N.R., Altundağ E.M., Süha Yalçın A. (2019). Combined Effects of Quercetin and Curcumin on Anti-Inflammatory and Antimicrobial Parameters in Vitro. Eur. J. Pharmacol..

