# Supplementary material for: Polyphenols as Potential β-Lactamase Inhibitors: An Integrated Computational and Experimental Study
Source: Molecules. 2025 Nov 15;30(22):4416. doi: 10.3390/molecules30224416 (PMC12655399; doi:10.3390/molecules30224416)
Supplement: Supplementary file 1 [file molecules-30-04416-s001.zip › molecules-3969511-supplementary.pdf]

Table S1: List the interacting amino acid residues involved in the ligand–protein interaction of the major compounds against three different targets.

	KPC-2		NDM-1		OXA-48-like	
	**Interacting Amino Acid**	**Type of Intermolecular Bond**	**Interacting Amino Acid**	**Type of Intermolecular Bond**	**Interacting Amino Acid**	**Type of Intermolecular Bond**
**Kaempferol**	Thr216, Ser130, Thr235	Conventional Hydrogen Bond, Pi-Donor Hydrogen bond, Pi-sigma	His122, His189, Cys208, Lys211, His250	Conventional Hydrogen Bond, Carbon Hydrogen Bond, Pi-Pi stacked Pi-Pi T-shaped	Ile102, Ser118, Tyr211	Conventional Hydrogen Bond, Pi-alkyl
**Quercetin**	Ser70, Trp105, Glu166, Thr216, Thr237, Thr235	Conventional Hydrogen Bond, Carbon Hydrogen, Pi-Pi stacked	His122, His189, Cys208, Lys211, His250	Conventional Hydrogen Bond, Carbon Hydrogen, Bond, Pi-Pi stacked Pi-Pi T-shaped	Ile102, Ser118, Tyr211	Conventional Hydrogen Bond, Pi-sigma
**Caffeic acid**	Trp105, Asn132, Asn170, Thr237, Thr235	Conventional Hydrogen Bond, Pi-Donor Hydrogen bond, Pi-Pi stacked	Asp124, His189, Cys208, Lys211, Asn220, His250	Conventional Hydrogen Bond, Van der Waals, Pi-Alkyl, Pi-Anion, Pi-Cation	Ser118, Thr209, Tyr211	Conventional Hydrogen Bond
**3,4-dihydroxybenzoic acid**	Lys73, Trp105, Asn132, Asn170, Thr235	Conventional Hydrogen Bond, Carbon Hydrogen, Pi-Donor Hydrogen bond	Asp124, His122, His189, Cys208s	Conventional Hydrogen Bond, Pi-Anion	Ser70, Val120, Arg214	Conventional Hydrogen Bond, Pi-alkyl

Table S2: Combined summary of Molecular docking score, RMSD, Rg, RMSF, and hydrogen bond analyses for β-lactamase–polyphenol complexes over 100 ns MD simulations.

Receptor	Enzyme–Ligand Complex	Docking Score (kcal/mol)	RMSD (stability)	Average Rg (nm) (compactness)	RMSF (flexibility)	H-Bonds (stability pattern)
KPC-2	KPC-2/Imipenem	-6.6	0.12 - 0.18	~1.80	0.05 – 0.30	1–4
	KPC-2/Quercetin	-8.3	0.12 -0.17	~1.80	0.05 – 0.20	1-3
	KPC-2/Kaempferol	-7.5	0.15 – 0.20	~1.81	0.05 – 0.25	2-4
	KPC-2/Caffeic acid	-6.3	0.10 -0.16	~1.79	0.05 – 0.22	1-3
	Kpc-2/3,4-dihydroxybenzoic acid	-5.8	0.09 - 0.21	~1.78	0.05 – 0.23	1-2
	NDM-1/Imipenem	-6.2	0.15 -0.25	~1.70	0.05 – 0.28	1-3
	NDM-1/Quercetin	-7	0.15 - 0.20	~1.67	0.05 – 0.20	2-3
	NDM-1/Kaempferol	-7	0.18 – 0.25	~1.69	0.05 – 0.23	2-3
	NDM-1/Caffeic acid	-5.3	0.14 – 0.22	~1.68	0.05 – 0.22	1-2
	NDM-1/3,4-dihydroxybenzoic acid	-5.2	0.13 – 0.31	~1.66	0.05 – 0.23	1-2
	OXA-48/Imipenem	-6.2	0.14 - 0.21	~1.81	0.05 – 0.22	1-3
	OXA-48/Quercetin	-8.3	0.15 – 0.22	~1.80	0.05 – 0.20	2-3
	OXA-48/Kaempferol	-8.2	0.16 – 0.25	~1.82	0.05 – 0.23	2-3
	OXA-48/Caffeic acid	-6.2	0.14 – 0.20	~1.80	0.05 – 0.21	1-2
	OXA-48/3,4-dihydroxybenzoic acid	-5.6	0.15 – 0.25	~1.79	0.05 – 0.21	1-2

Figure S1: Effect of the combination of cefotaxime with the four polyphenols on *K. pneumonia, P. aeruginosa,* and *E. coli* isolates.

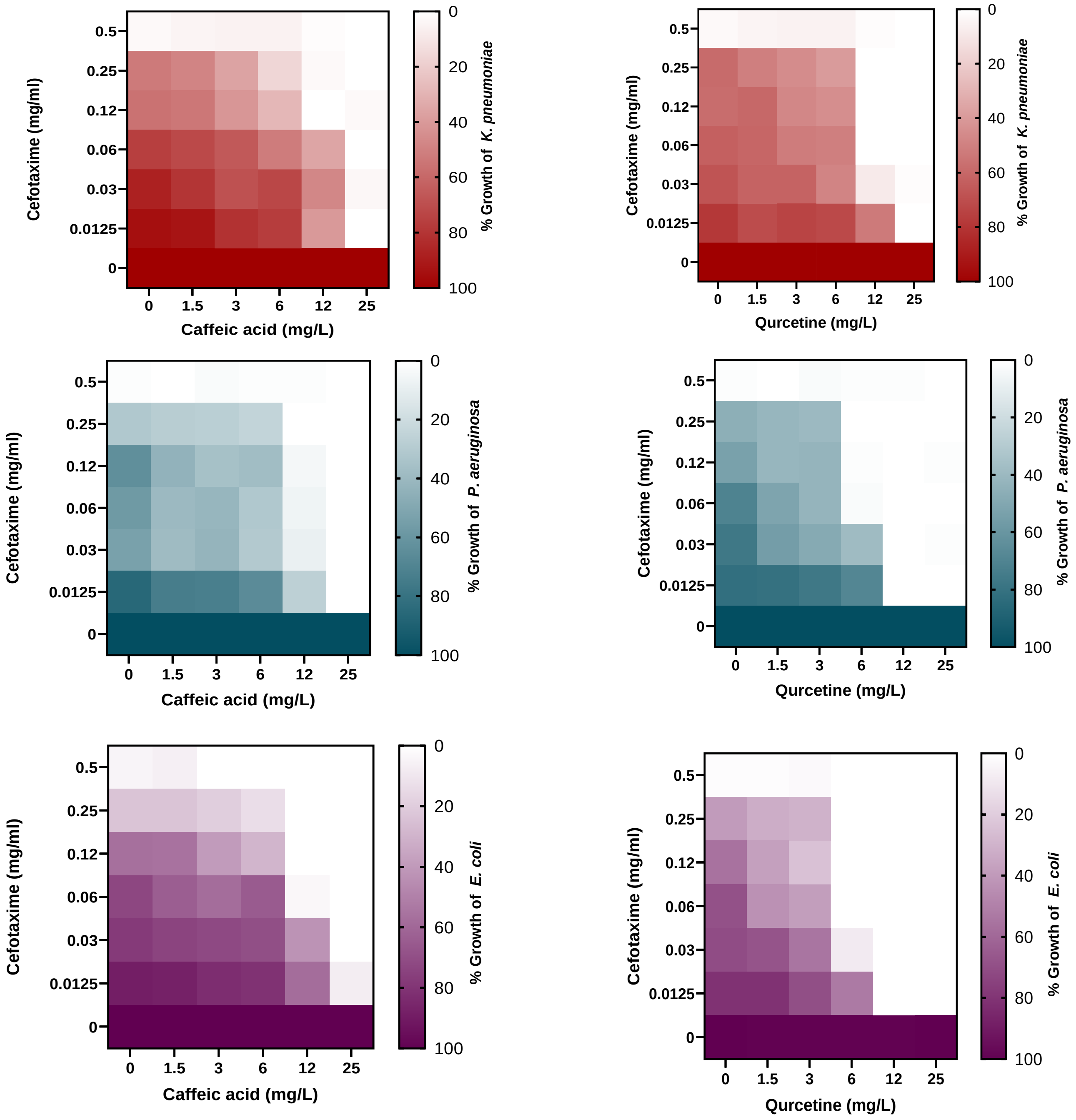

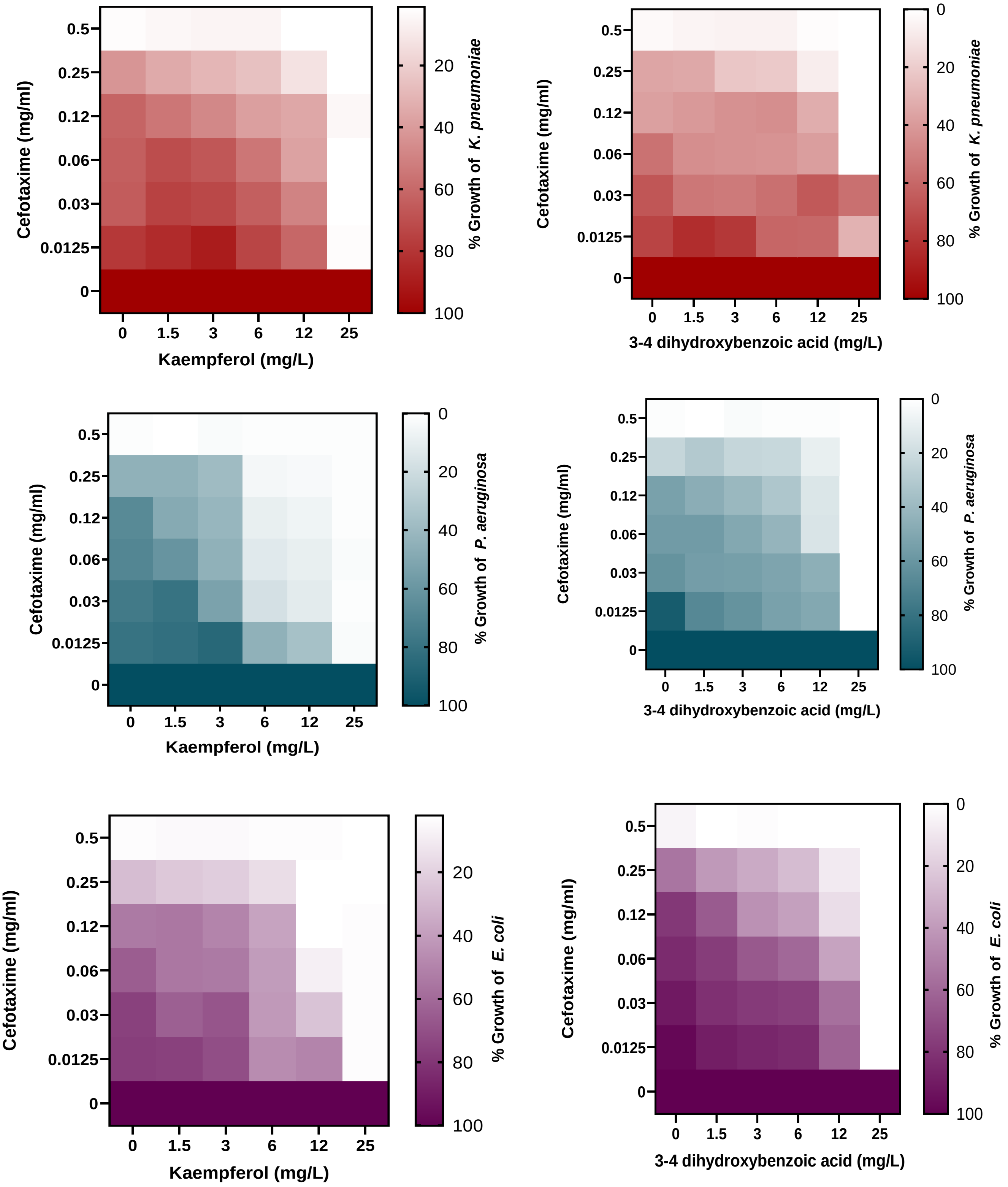
Cefotaxime (mg/ml)
0.5
0.25
0.12
0.06
0.03
0.0125
0
0 1.5 3 6 12 25
Kaempferol (mg/L)
% Growth of K. pneumoniae
20 40 60 80 100
3-4 dihydroxybenzoic acid (mg/L)
0 20 40 60 80 100
% Growth of P. aeruginosa
% Growth of E. coli
